# Comprehensive Molecular Analyses of a Macrophage-Related Gene Signature With Regard to Prognosis, Immune Features, and Biomarkers for Immunotherapy in Hepatocellular Carcinoma Based on WGCNA and the LASSO Algorithm

**DOI:** 10.3389/fimmu.2022.843408

**Published:** 2022-05-27

**Authors:** Tao Wang, Liqun Dai, Shu Shen, Yi Yang, Ming Yang, Xianwei Yang, Yiwen Qiu, Wentao Wang

**Affiliations:** ^1^Department of Liver Surgery and Liver Transplantation Center, West China Hospital of Sichuan University, Chengdu, China; ^2^State Key Laboratory of Biotherapy and Cancer Center, West China Hospital, Sichuan University, Chengdu, China; ^3^Department of Thyroid Surgery, Sichuan Provincial People’s Hospital, University of Electronic Science and Technology of China, Chengdu, China

**Keywords:** macrophage-related genes, hepatocellular carcinoma (HCC), immunity, prognosis, immune drug response

## Abstract

Macrophages have been reported to exert a crucial role in hepatocellular carcinoma (HCC). This study aimed to explore the macrophage-related genes and establish a macrophage-related signature (MRS) model to predict the overall survival (OS) of patients with HCC based on these genes’ expression. We screened the macrophage-related gene module by weighted gene coexpression network analysis (WGCNA), the least absolute shrinkage and selection operator (LASSO) Cox regression analysis was utilized for further selection, and the selected genes were entered into stepwise regression to develop the MRS model, which was further validated in the Gene Expression Omnibus (GEO) and International Cancer Genome Consortium (ICGC) datasets. We analyzed the biological phenotypes associated with macrophages in terms of functional enrichment, tumor immune signature, and tumor mutational signature. The patient’s response to immunotherapy was inferred by the tumor immune dysfunction and exclusion (TIDE) score, the immunophenotype score (IPS), and the IMvigor210 dataset. A novel MRS model was established based on the LASSO regression coefficients of the genes *PON1*, *IL15RA*, *NEIL3*, *HILPDA*, *PFN2*, *HAVCR1*, *ANXA10*, *CDCA8*, *EPO*, *S100A9*, *TTK*, *KLRB1*, *SPP1*, *STC2*, *CYP26B1*, *GPC1*, *G6PD*, and *CBX2*. In either dataset, MRS was identified as an independent risk factor for OS in HCC patients. Additionally, our research indicated that a high-risk score in the MRS model was significantly correlated with tumor staging, pathological grade, tumor–node–metastasis (TNM) stage, and survival. Several genes of the human leukocyte antigen (HLA) family and immune checkpoints were highly expressed in the high-risk group. In addition, the frequency of tumor mutations was also higher in the high-risk group. According to our analyses, a higher risk score in the MRS model may predict a better response to immunotherapy.

## Introduction

Liver cancer is a highly heterogeneous malignancy that is considered the fourth most common cause of cancer-related deaths worldwide, with more than half of all new cases and deaths from liver cancer occurring in China each year (1–4). Hepatocellular carcinoma (HCC), the most common histological type of liver cancer, has a poor prognosis. With the progression of modern medical science and technology, great strides have been made in the treatment of HCC. However, because the clinical symptoms of early HCC are not obvious, 70%–80% of patients are in the advanced stage when they are diagnosed. Currently, the overall survival (OS) of HCC treatment is still not ideal. Therefore, it is urgent to clarify the molecular mechanism of tumor progression and develop new therapeutic target agents to prolong the survival time of HCC patients.

HCC is a typical immunogenic cancer that appears almost exclusively in the presence of chronic inflammation (5). Immune imbalance in the tumor microenvironment (TME) is one of the important landscapes of HCC (6). The TME, which is the “soil” for tumor growth and survival, is one of the important determinants influencing the occurrence and progression of tumor cells (seeds) as well as the response to various treatments. Macrophages, being an important part of the immune microenvironment, play an irreplaceable role in the body’s innate immunity and acquired immunity (7). Tumor-associated macrophages (TAMs) are the most abundant infiltrating immune cells in the tumor microenvironment, where they perform a broad repertoire of functions in HCC *via* their diverse phenotypes. TAMs are usually divided into different subsets, including the M1 type (classical activated macrophages) and the M2 type (replacement of activated macrophages) (8, 9). In the early stage of tumor development, TAMs mainly have an M1 pro-inflammatory phenotype and mediate immune responses that inhibit tumor growth. As the tumors develop, TAMs gradually transform to the M2 type function phenotype, which in turn promotes their participation in immunosuppression and tumor angiogenesis (10, 11). There is accumulating evidence that the tumorigenesis, progression, and metastasis of tumors are influenced by dynamic changes in macrophage phenotypes (12). In addition, previous studies found that TAMs can attract immunosuppressive cells (including Treg cells and myeloid-derived suppressor cells) to the tumor site by producing a variety of chemokines and can induce monocytes to express the costimulatory molecule programmed death ligand (PD)-L1 to inhibit the cytotoxic T-cell response (13–15). TAMs can also produce angiogenic factors and express matrix metalloproteinases to induce tumor angiogenesis. In this regard, elucidating the relevant genes and characteristics of TAMs and identifying biomarkers related to macrophage infiltration are essential for the treatment and prognosis of HCC, as they will help us monitor the HCC immunotherapy response and further explore the mechanism of immune infiltration. However, so far, few studies have comprehensively and systematically described the characteristics of the immune microenvironment and the immune cell types of HCC, particularly its macrophages. Thus, we performed this comprehensive systematic study to identify macrophage-related genes and construct coexpression networks of macrophages using the weighted gene coexpression network analysis (WGCNA) approach, and then established a macrophage-related risk signature (MRS) to test its prognostic value in predicting the prognosis of HCC patients and their response to chemotherapy and immunotherapy. The results of this study will provide insights into the impact of macrophages on HCC and help enhance the effectiveness of individualized treatment for HCC patients.

## Materials and Methods

### Datasets and Sample Extraction

**Figure 1:**
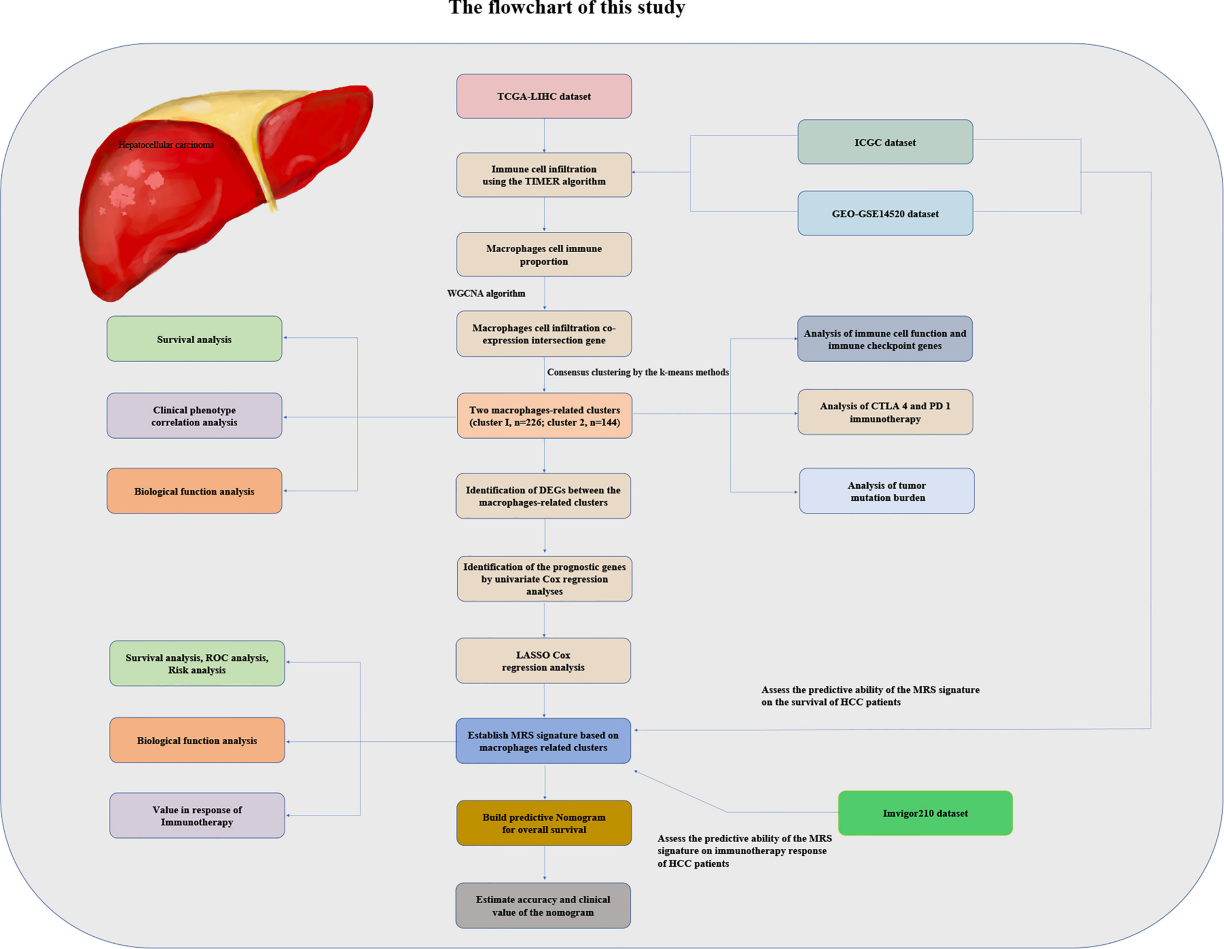
The flowchart of this study.

We obtained HCC patients’ RNA sequencing (RNA-seq) expression data, genomic mutation data, and accompanying clinical data from The Cancer Genome Atlas (TCGA) database (https://portal.gdc.cancer.gov/repository). Similarly, the RNA-seq expression data and matched clinicopathologic information were also downloaded from the GSE14520 database (16) of the Gene Expression Omnibus (GEO) repository (https://www.ncbi.nlm.nih.gov/gds/) and from the International Cancer Genome Consortium (ICGC) database (https://dcc.icgc.org/projects/LIRI-JP). We utilized the transcriptome data and clinical data of the IMvigor210 dataset of patients with metastatic urothelial cancer treated with anti-PD-L1 drugs obtained from the IMvigor210CoreBiologies R package to verify whether the risk model we established could predict the effectiveness of immunotherapy. The flowchart of the present study design is shown in **Figure 1**.

### Macrophage Coexpression Network Construction

The TIMER algorithm was utilized to estimate the relative proportion of infiltrating immune cells in HCC samples in each cohort (17). We used WGCNA which can convert coexpression correlation into connection weights or topological overlap values (18), to identify coexpressed genes in macrophages. Network type was set as the “unsigned” type. We used standard deviation (SD > 50%) to screen highly variable genes in the WGCNA expression data. The topological overlap matrix was employed to determine the connectivity and dissimilarity of the coexpression network established with the appropriate soft thresholding parameter (**Figure 1**). The dynamic hybrid cut method (a bottom-up algorithm) was used to identify coexpressed gene modules. A hierarchical clustering tree was established using dynamic hybrid cutting. Each leaf on the tree represents a single gene, and genes with similar expression data or similar functions are close together and form a branch of the tree, representing a gene module. We used Pearson’s test to calculate the correlation between module eigengenes (MEs) and macrophages. When *P <*0.05, the module was considered to be significantly related to macrophages. In this way, a set of genes related to the proportion of macrophages with similar functions was identified in the same module. Subsequently, we took the intersection of the genes of the modules most related to macrophages in the three cohort datasets and displayed them in the form of a Venn diagram.

### Consensus Clustering Analysis

Based on the expression of macrophage-related intersection genes, unsupervised hierarchical clustering was applied to classify HCC patients into the optimal number of clusters using the “ConsensusClusterPlus” package of R (19).

### Calculation of Tumor Mutation Burden

Tumor mutational burden (TMB) in each tumor sample refers to the number of mutated bases per million bases, which include missense mutations, nonsense mutations, frameshift mutations, and so on. We computed the TMB values from the number of variants out of the total length of the human exons (38 million) in each sample by using Perl scripts. Waterfall plots were generated using the “maftools” R package to assess the number of somatic point mutations in each HCC sample and to illustrate the relationship between TMBs and risk groups (20). We calculated the copy number variation (CNV) frequencies and displayed the above results in a lollipop chart. The “RCircos” package of R software was utilized to visualize the location of these genes on the chromosomes.

### Functional Enrichment Analysis

Gene ontology (GO) and Kyoto Encyclopedia of Genes and Genomes (KEGG) were used to assess the biological roles of the prognostic candidates by the “clusterProfiler” R package. Gene set enrichment analysis (GSEA) software (version 4.1.0) was used to compare the biological processes that were significantly different between the low- and high-risk groups.

### Immunogenomic Landscape Analysis

In order to investigate the difference in the TME among macrophage-related clusters, the stromal score, immune score, estimate score, and tumor purity were estimated using the “ESTIMATE” R package based on the results of single-sample gene set enrichment analysis (ssGSEA) (21). ssGSEA with the “gsva” package was implemented to calculate the activity scores of the activities of immune-related pathways (22). Immune infiltration and functions were compared between the different groups by using the two-sample Wilcoxon test. We further compared the expression of common immune checkpoint inhibitor (ICI) and human leukocyte antigen (HLA) genes between different clusters. Additionally, we acquired an overview of the immune subtypes of HCC patients from the TGGA database from UCSC-Xena (https://xenabrowser.net/datapages/) (23). We made comparisons of the immune subtypes between different risk groups based on macrophage-related clusters using the package “RColorBrewer.”

### Prediction of Immunotherapy Response

The immunophenoscore (IPS) of HCC samples was obtained from the LIHC project of The Cancer Immunome Atlas (TCIA, https://tcia.at/), which can predict the response to immunotherapies including CTLA4 and PD-1 blockers (24). The IPS score was normalized to a range of 0 to 10, where a higher IPS score represents higher immune reactivity. The tumor immune dysfunction and exclusion (TIDE) was used to predict the potential immune checkpoint blockade responses in HCC. A lower TIDE score represents a better response to immunotherapy. In addition, we utilized the IMvigor210 dataset to validate the links between the risk signature and immunotherapy.

The TISMO (tismo.cistrome.org) database was used to compare gene expression levels across groups of different responses to immune checkpoint blockers (ICBs) in syngeneic mouse models.

### Identification of a Prognostic Macrophage-Related Score Model for HCC

Based on the RNA-seq results, differentially expressed genes (DEGs) between cluster 1 and cluster 2 were identified. Then, we used univariate Cox regression analysis to identify the genes with good predictive ability for prognosis. To further narrow down the candidate prognosis-related genes and synthetically estimate the significance values, the LASSO Cox regression algorithm (R package “glmnet”) was utilized to identify the variation in regression coefficients of the prognostic genes and select the optimal and minimal criteria of the penalization parameter (*λ*) using 10-fold cross-validation. Immunohistochemistry data from the Human Protein Atlas (HPA) database confirmed the expression of these model genes in HCC. All HCC patients were grouped into high- and low-risk groups by the median risk score value. The difference in expression between the identified genes by the LASSO Cox regression algorithm and the distribution pattern between clinicopathological characteristics and risk groups are displayed in the form of heatmaps using the R package “pheatmap.” Principal component analysis (PCA) and t-distributed random neighborhood embedding (t-SNE) analysis with the R package “Rtsne” were applied to gauge the discriminative ability of the predictive model. The area under the time-dependent receiver-operating characteristic curve (AUROC) was used to appraise the predictive ability of the risk groups identified above. A risk curve was drawn to explore the difference in survival status between different risk groups of patients. Then, the accuracy of the risk score model was validated in the GSE14520 and ICGC datasets using the same method.

### Construction and Validation of the Clinical Prognostic Model

To evaluate whether risk score is an independent risk factor affecting the survival of HCC patients, univariate and multivariate analyses were conducted on the TCGA, GSE14520, and ICGC datasets. Similarly, decision curve analysis (DCA) was carried out to determine the clinical application value of the risk score model by calculating the net benefits at each risk threshold (25). A nomogram integrating sex, grade, age, stage, and risk signature for survival prediction was then established using the “regplot” package. The calibration curve was drawn to evaluate the predictive accuracy of the nomogram.

### Analysis of Chemotherapeutic Drug Sensitivity Between Different MRS Groups

In view of the lack of biomarkers that can accurately predict chemotherapeutic drug sensitivity in HCC patients, we conducted drug susceptibility analyses through the “pRRophetic” and “ggplot2” packages to compare the half-maximal inhibitory concentration (IC_50_) of various chemotherapeutic drugs for HCC between the high- and low-risk groups using the Wilcoxon signed-rank test (26).

### Statistical Analysis

All statistical analyses in our study were executed by R software (version 4.1.0, https://www.r-project.org/). For comparison of continuous variables between two groups, the independent Student’s *t*-test was performed, and the Wilcoxon rank-sum test was utilized to compare non-normally distributed variables. The chi-squared test was used for the comparison of categorical variable data between two groups. The *P*-value for statistically significant differences was set to 0.05, unless otherwise stated in the manuscript.

## Results

### Identification of Hub Modules Associated With Macrophage Infiltration in HCC and Enrichment Analysis

**Figure 2:**
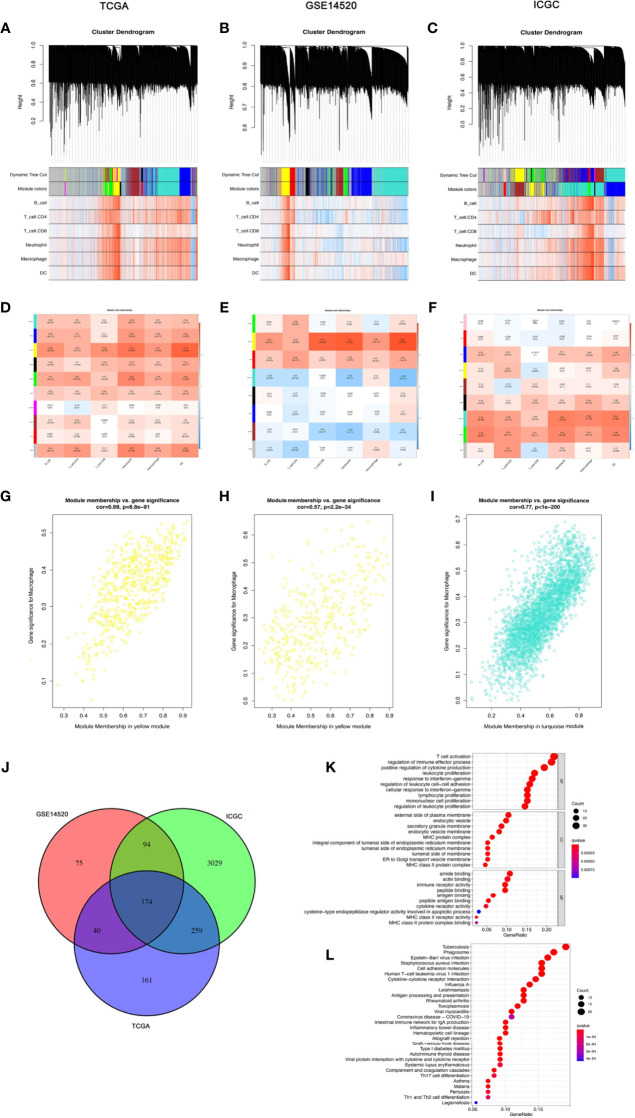
The coexpression network established using weighted gene coexpression network analysis methods based on the **(A)** LIHC RNA-seq profiles from the TCGA-LIHC database, **(B)** GSE14520 dataset, and **(C)** the International Cancer Genome Consortium portal database. Heatmap demonstrating the correlation between module eigengenes and macrophages in the **(D)** TCGA-LIHC dataset, **(E)** GSE14520 dataset, and **(F)** International Cancer Genome Consortium portal dataset. **(G)** The yellow module had the strongest correlation with macrophage cell proportions in the TCGA-LIHC dataset (Cor = 0.69, *P* = 8.8e−91). **(H)** The yellow module had the strongest correlation with macrophage cell proportions in the GSE14520 cohort (Cor = 0.57, *P* = 2.2e−34). **(I)** The turquoise module had the strongest correlation with macrophage cell proportions in the ICGC (Cor = 0.77, *P* < 1e−200). **(J)** Venn diagram displaying the macrophage-related selected intersection genes from different datasets. **(K, L)** Gene ontology (GO) and Kyoto Encyclopedia of Genes and Genomes (KEGG) analyses of macrophage-related intersecting genes.

In the TCGA-LIHC cohort dataset, among the 10 modules, the yellow module was highly correlated with macrophages (*R*^2^ = 0.51, *P* = 1e−26). In the GSE14520 cohort dataset, among the eight modules, the yellow module was highly correlated with macrophages (*R*^2^ = 0.51, *P* = 3e−26). In the ICGC cohort dataset, among the nine modules, the turquoise module was highly correlated with macrophages (*R*^2^ = 0.58, *P* = 1e−22). Given that our study focused on macrophages, the yellow module from the TCGA-LIHC dataset and the GSE14520 dataset and the turquoise module from the ICGC dataset were identified as the hub module, as shown in **Figure 2A**.

Subsequently, 174 macrophage coexpressed genes were identified from the intersection of the above modules (**Figure 2**). We compared the expression levels of all 174 macrophage coexpressed genes between tumor tissues and normal samples, and we identified 69 differentially expressed macrophage coexpressed genes (|log_2_ FC|  ≥ 0.585, all *P* < 0.05), as shown in **Supplementary Figure S1**. Then, the potential interactions among these differentially expressed macrophage coexpressed genes were analyzed by the PPI network (**Supplementary Figure S1**). The correlation network containing all differentially expressed macrophage coexpressed genes is presented in **Supplementary Figure S1**. GO enrichment analysis and KEGG pathway analysis were then applied to the genes lying in the intersection of the above three cohort datasets (**Figure 2K****)**.

### The Biological Characteristics of Each Macrophage-Related Cluster

**Figure 3:**
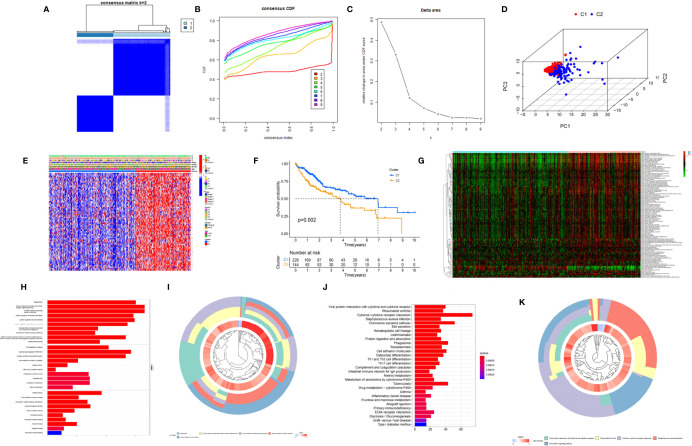
Cluster analysis of intersection genes related to macrophages in the TCGA cohort. **(A)** The LIHC dataset in the TCGA cohort was divided into two distinct clusters when *k* = 2. **(B)** Consensus clustering cumulative distribution function (CDF) for *k* = 2 to 9. **(C)** Relative change in the area under the CDF curve for *k* = 2 to 9. **(D)** The 3D PCA plots showed the cluster could distinguish hepatocellular carcinoma (HCC) patients based on the expression profiles of the LIHC dataset. **(E)** Heatmap of distribution of clinicopathological variables between different cluster groups. **(F)** The Kaplan–Meier curve survival analysis between different cluster groups. **(G)** The heatmap displaying the biological processes in distinct macrophage-related clusters analyzed by gene set variation analysis. **(H)** The bar plot and **(I)** the cluster plot of the GO pathways of enriched differentially expressed genes between different cluster groups. **(J)** The bar plot and **(K)** the cluster plot of the KEGG pathways of enriched differentially expressed genes between different cluster groups. *P < 0.05; **P < 0.01.

To further explore the heterogeneity of macrophages between tumors in HCC, based on the expression level of 174 macrophage coexpresssed genes, unsupervised consensus analysis was employed to divide these 174 macrophage coexpression genes in TCGA-LIHC into two different subtypes with different molecular and clinical characteristics, including 226 samples in macrophage-related cluster 1 and 144 samples in macrophage-related cluster 2 (**Figures 3A–C**). 3D PCA showed that the two clusters could be well-distinguished by the macrophage-related coexpressed genes (**Figure 3D**). The heatmap indicated the distribution of clinicopathological characteristics in cluster 1 and cluster 2. We found significant differences in tumor stage status and pathological grade in the two clusters (**Figure 3E**). Furthermore, we found that patients in cluster 1 had significantly better OS than those in cluster 2 (**Figure 3F**). GSVA enrichment analysis showed that there were differences in the enriched pathways between cluster 1 and cluster 2 (**Figure 3G**). In total, 2,216 DEGs between the different clusters were identified. GO enrichment and KEGG pathway analyses were then run on the above DEGs, as shown in **Figures 3H–K**.

**Figure 4:**
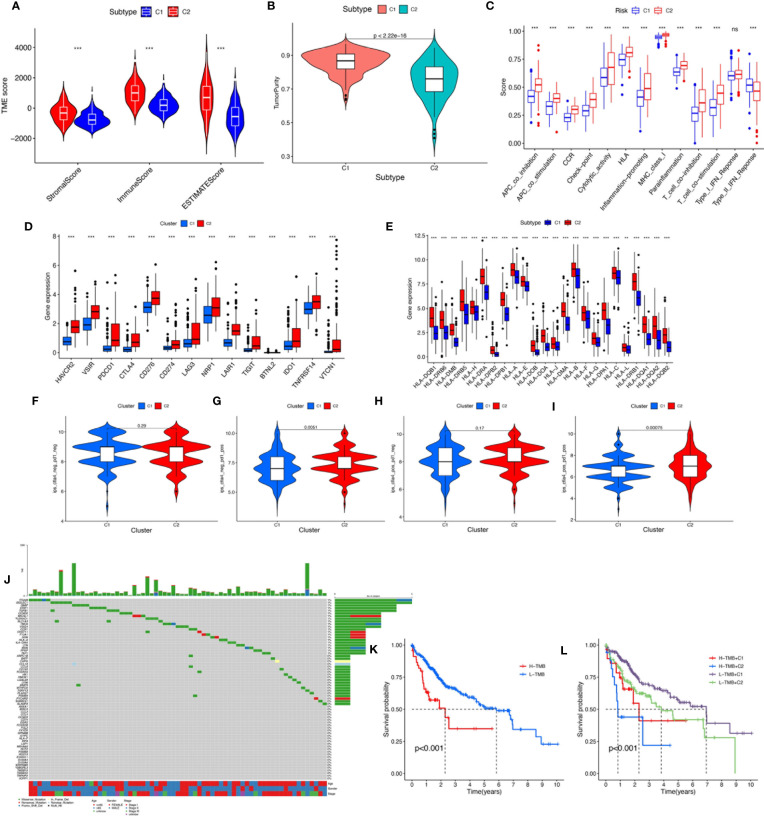
**(A)** The comparisons of stromal score, immune score, and estimate score between different clusters. **(B)** The comparisons of tumor purity between different clusters. **(C)** The boxplot illustrating the difference in immune-related functions between different clusters. **(D)** The boxplot displaying the difference in immune checkpoint genes between different clusters. **(E)** The boxplot displaying the difference in HLA expression between different cluster groups. **(F)** CTLA4^−^_PD1^−^, **(G)** CTLA4^−^_PD1^+^, **(H)** CTLA4^+^_PD1^−^, and **(I)** CTLA4^+^_PD1^+^. The comparison of immunophenoscore (IPS) between different cluster groups. **(J)** Somatic mutation landscape of macrophage-related differentially expressed genes was presented by a waterfall map. **(K)** The Kaplan–Meier curve survival analysis between the high- and low-TMB groups. **(L)** The Kaplan–Meier curve survival analysis for HCC patients stratified by both TMB groups and clusters. ns, not significant; **P < 0.01; ***P < 0.001.

To verify the reliability of the above cluster scheme and compare the tumor immune microenvironment and activities of immune-related pathways between the two groups, we calculated the immune score, estimate score, stromal score, and tumor purity based on the gene expression profile of each HCC sample using the ESTIMATE algorithm. The immune score, stromal score, and estimate score in cluster 1 were significantly lower than those in cluster 2 (**Figure 4**), while tumor purity in cluster 1 was significantly higher than that in cluster 2 (**Figure 4**). These results suggested that the above two clusters had completely different TME infiltration characteristics. There were marked differences in immune cell-related functions between the two clusters. The type II IFN response was enriched in cluster 1, but others were enriched in cluster 2. Finally, we compared the expression of ICI genes and HLA genes between clusters. Immune checkpoint genes (*HAVCR2*, *VSIR*, *PDCD1*, *CTLA4*, *CD276*, *CD274*, *LAG3*, *NRP1*, *TIGHT*, *BTNL2*, *IDO1*, *TNFRSF14*, and *VTCN1*) were highly expressed in cluster 2, while the HLA-related genes were highly expressed in cluster 2 (**Figure 4D****)**. To further test the ability of the macrophage-associated clusters to predict the ICI response, IPS analysis was carried out to determine the immunotherapeutic sensitivity of HCC patients, respectively. As shown in **Figure 4F**, the IPS–PD1 blocker score, the IPS–CTLA4 score, and the PD1 blocker score in cluster 2 were significantly higher than those in cluster 1, which indicated a more immunogenic phenotype in cluster 2, so HCC patients in cluster 2 might benefit from immunotherapy.

TMB, also known as non-synonymous variation, is strongly associated with immune cell infiltration and immune response (27). Somatic mutation data of HCC patients were obtained and TMB scores were computed. The waterfall plot showed mutations of the macrophage-related DEGs (**Figure 4**). We divided the HCC patients into the low-TMB and high-TMB groups around the optimal cutoff value. The Kaplan–Meier curve indicated that the prognosis of patients with high TMB was poorer than that of patients with low TMB. In addition, given the presence of a potential correlation between macrophage-related clusters and TMB, we performed the stratification analysis and found that the cluster combined with the TMB risk group can better predict the prognosis of HCC patients (**Figure 4K****)**.

### Development and Validation of the MRS Models

**Figure 5:**
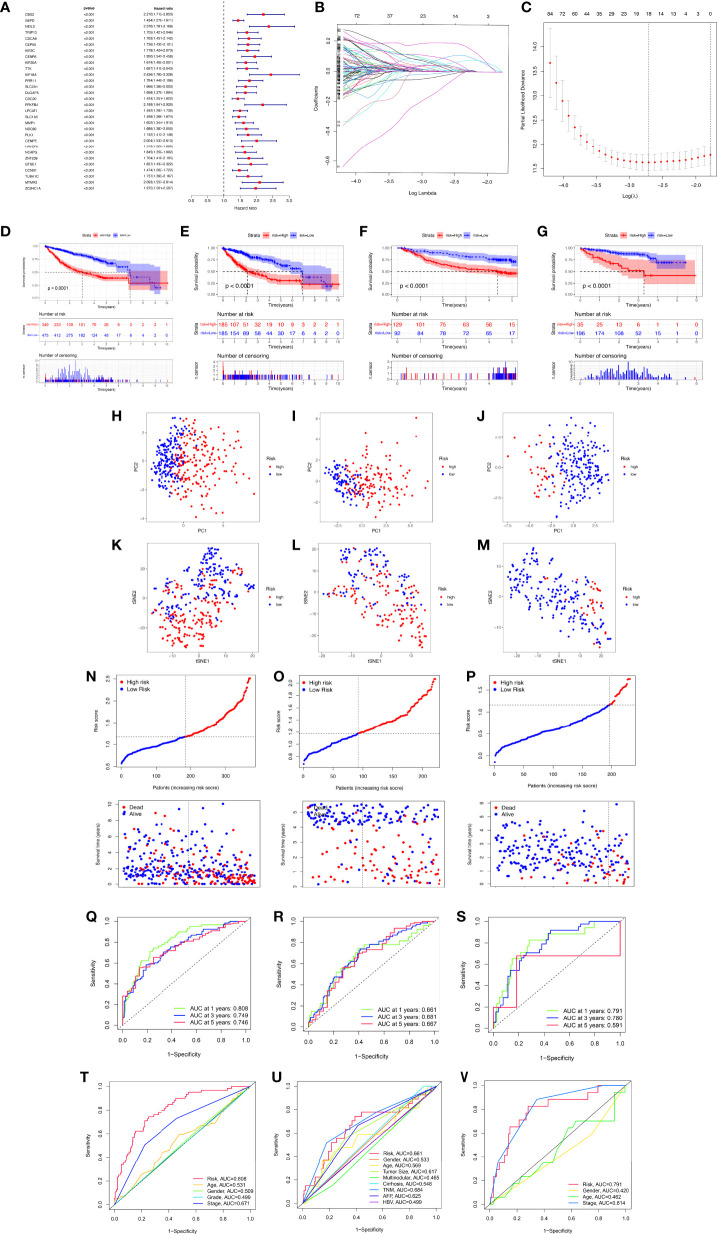
Development and validation of the macrophage-related signature (MRS) signature based on macrophage-related clusters. **(A)** The forest plot displaying the HR (95% CI) and *P*-values for selected differentially expressed genes between different clusters using the univariate Cox regression analysis (top 30, according to *P*-value). **(B)** Eighteen gene expression signatures based on macrophage-related clusters were selected by the LASSO Cox models. **(C)** Cross-validation for tuning parameter selection in the LASSO model. **(D)** The Kaplan–Meier curve survival analysis for HCC patients stratified by MRS signature groups in the three cohort datasets (TCGA-LIHC, GSE14520, ICGC). **(E)** The Kaplan–Meier curve survival analysis for HCC patients stratified by MRS signature groups in the TCGA-LIHC dataset. **(F)** The Kaplan–Meier curve survival analysis for HCC patients stratified by MRS signature groups in the GSE14520 dataset. **(G)** The Kaplan–Meier curve survival analysis for HCC patients stratified by MRS signature groups in the ICGC dataset. **(H–K)** Principal component analysis (PCA) between the high- and low-risk groups in the TCGA-LIHC, GSE14520, and ICGC datasets. **(K–M)** t-distributed stochastic neighbor embedding (t-SNE) analysis between the high- and low-risk groups in the TCGA-LIHC, GSE14520, and ICGC datasets. **(N–P)** The risk score distribution and survival status distribution of HCC patients in the two risk groups from the TCGA-LIHC, GSE14520, and ICGC datasets. **(Q–S)** ROC analysis for OS prediction including 1, 3, and 5 years of HCC patients in the TCGA-LIHC, GSE14520, and ICGC datasets. **(T–V)** ROC curve analysis compares the predictive power of the MRS signature and other clinicopathological indicators in the TCGA-LIHC, GSE14520, and ICGC datasets.

To better apply these subtypes to the clinical treatment of HCC and calculate the specific risk score of each HCC patient, subsequently, we compared the DEGs between the two clusters and established a specific risk scoring model based on macrophage-related clusters (**Figure 5**). Subsequently, based on the obtained gene expression profiles, an 18-gene risk score model based on macrophage-related clusters was established for each patient based on the personalized gene level using LASSO Cox regression analyses (**Supplementary Figure S2**), named the macrophage-related risk score signature (MRS) (**Figures 5B, C****)**. We utilized the Kaplan–Meier curve analysis to compare the effects of high and low expression of the above-selected genes on long-term survival (**Supplementary Figure S3**). Kaplan–Meier analysis revealed that patients in the high-risk group had significantly poorer OS than those in the low-risk group, as shown in **Figures 5D–G**. PCA and t-SNE analysis indicated that the MRS model showed good discrimination (**Figures 5H–M**). Risk curve analysis indicated that the high-risk group had higher mortality and shorter survival time (on the right side of the dotted line). The number of patients in the high-risk group grew and death events increased with increasing risk score (**Figures 5N–P**). The AUCs for 1-, 3-, and 5-year OS were 0.808, 0.749, and 0.746, respectively, in the TCGA cohort (**Figure 5Q**). Consistent with the results in the TCGA cohort, the AUC values for 1-, 3-, and 5-year OS were 0.661, 0.681, and 0.667, in the GSE14520 cohort, respectively (**Figure 5R**). The AUC values for 1-, 3-, and 5-year OS were 0.791, 0.780, and 0.591 in the ICGC cohort, respectively (**Figure 5S**). Furthermore, the AUC value for MRS based on macrophage-related clusters was significantly higher than those for age, sex, tumor stage, and pathological stage in the TCGA-LIHC cohort. Meanwhile, the MRS model revealed a good predictive ability that was not inferior to the predictive ability of other clinicopathological factors in the GSE14520 and ICGC cohorts (**Figures 5T–V**).

Immunohistochemical results from the HPA database were utilized to further assess the expression of genes from the MRS model in HCC. Compared with adjacent normal liver tissues, the protein expression levels of genes (*PON1*, *KLRB1*, *ANXA10*) in HCC tissues decreased significantly, while the protein expression levels of *IL15RA*, *HILPDA*, *HAVCR1*, *G6PD*, *CDCA8*, and *CBX2* in HCC tissues increased. In addition, these data showed significant differences in *PON1*, *KLRB1*, *ANXA10*, *IL15RA*, *HILPDA*, *HAVCR1*, *G6PD*, *CDCA8*, and *CBX2* between HCC and normal liver tissues, and this trend was the same as in the model (**Supplementary Figure S4**).

### Independent Prognostic Value of the MRS Model and Construction of the Nomogram

**Figure 6:**
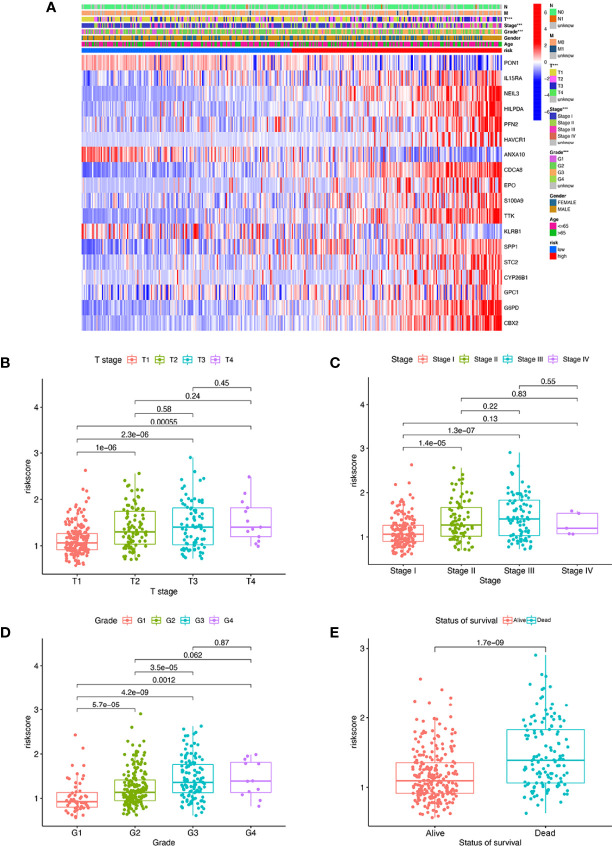
The MRS signature based on macrophage-related clusters was associated with the clinicopathological characteristics of patients with HCC in the TCGA-LIHC dataset. **(A)** Heatmap for the MRS signature based on macrophage-related clusters and clinicopathological manifestation. **(B)** Boxplot of risk score based on macrophage-related clusters in HCC patients with different stages. **(C)** Boxplot of risk score based on macrophage-related clusters in HCC patients with different tumor stages. **(D)** Boxplot of risk score based on macrophage-related clusters in HCC patients with different pathological grades. **(E)** Boxplot of risk score based on macrophage-related clusters in HCC patients with different status of survival. ***P < 0.001.

**Figure 7:**
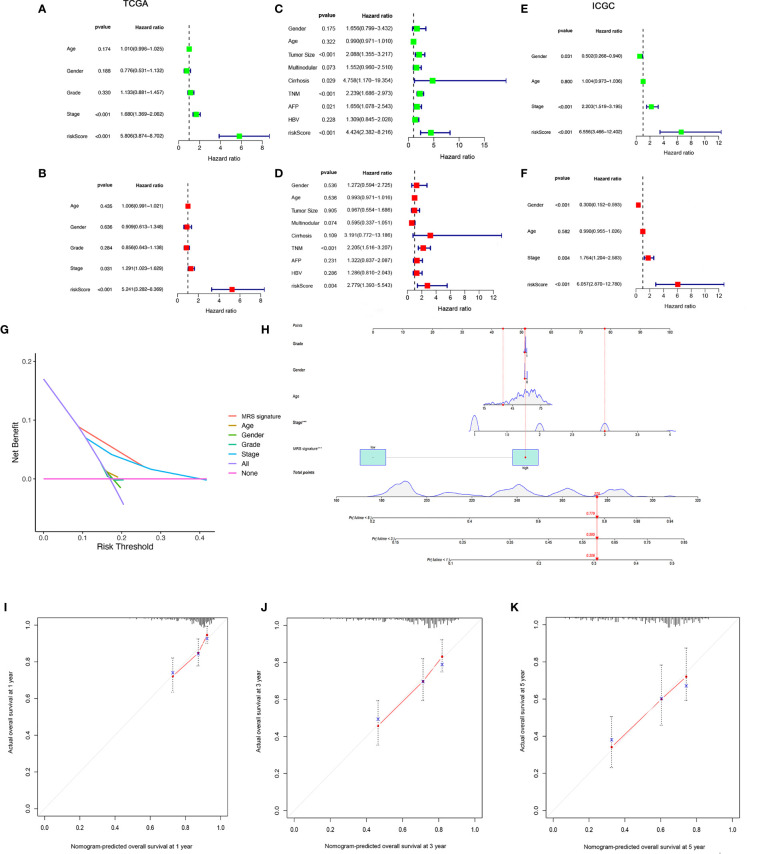
Establishment and assessment of the nomogram for survival prediction. **(A, B)** Univariate and multivariate Cox regression analyses showed that risk score based on macrophage-related clusters is an independent prognostic factor affecting the prognosis of HCC patients in the TCGA-LIHC dataset. **(C, D)** Univariate and multivariate Cox regression analyses showed that risk score based on macrophage-related clusters is an independent prognostic factor affecting the prognosis of HCC patients in the GSE14520 dataset. **(E, F)** Univariate and multivariate Cox regression analyses showed that risk score based on macrophage-related clusters is an independent prognostic factor affecting the prognosis of HCC patients in the ICGC dataset. The decision curve analysis of the 1-year overall survival in the TCGA-LIHC dataset **(G)**. **(H)** The nomogram combining risk score based on macrophage-related clusters and other clinicopathological parameters was developed to predict 1-, 3-, and 5-year survival. Calibration curves showing the predictions of the nomogram that we established for 1- **(I)**, 2- **(J)**, and 3-year **(K)** overall survival. ***P < 0.001.

We found that there were significant correlations between risk score and pathologic grade, T status, and tumor stage in the TCGA-LIHC cohort (**Figure 6**, *P* < 0.05). There were positive correlations between risk score and AFP level, cirrhosis, tumor size, and tumor stage in the GSE14520 cohort (**Supplementary Figures S5A–G**). The risk score in stage IV was significantly higher than that in stages I and II, and the risk score in the death group was significantly higher than that in the alive group in the ICGC cohort (**Supplementary Figures S5H–J**). In addition, univariate and multivariate Cox regression analyses indicated that the MRS was an independent factor predicting survival, as shown in **Figures 7A–F**. The decision curve analysis indicated that the MRS had a higher clinical net benefit than other clinicopathological characteristics in the TCGA-LIHC cohort (**Figure 7G**). Subsequently, based on the results of the stepwise Cox regression model, we further constructed a clinically adaptable nomogram with the MRS and other clinicopathological characteristics to provide a visual way to predict the 1-, 3-, and 5-year survival with HCC (**Figure 7H**). Our nomogram exhibited better accuracy in predicting both short- and long-term survival. The calibration plot of the nomogram showed excellent concordance between the prediction by the nomogram and the actual observation probabilities (**Figures 7I–K**). The above findings suggest that the nomogram we established has good prognostic value for patients with HCC.

### Functional Analyses Based on the MRS Model

**Figure 8:**
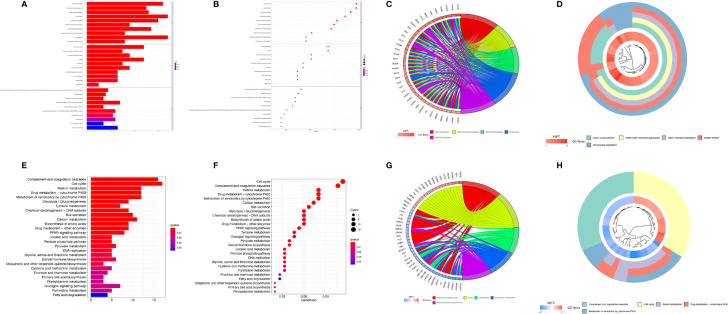
Functional analysis based on differentially expressed genes (DEGs) between the high-risk and low-risk groups from the TCGA-LIHC dataset. The bubble plot **(A)**, bar plot **(B)**, circular plot **(C)**, and cluster plot **(D)** of the GO pathways enriched for the DEGs between the high-risk and low-risk groups according to the risk score based on macrophage-related clusters. The bubble plot **(E)**, bar plot **(F)**, circular plot **(G)**, and cluster plot **(H)** of KEGG pathways enriched for the DEGs between the high-risk and low-risk groups according to risk score based on macrophage-related clusters.

To further analyze the differences in the gene functions and involved pathways between the subgroups classified by MRS, 220 DEGs between the low- and high-risk groups were identified. GO enrichment analysis and KEGG pathway analysis were then performed on these DEGs. GO pathway enrichment analysis revealed that the DEGs were mainly concentrated in “mitotic nuclear division,” “mitotic sister chromatid segregation,” “sister chromatid segregation,” and “nuclear division” in the biological process category. The KEGG analysis results showed that the DEGs were mainly enriched in pathways associated with “complement and coagulation cascades,” “cell cycle,” “retinol metabolism,” “drug metabolism-cytochrome P450”, and “metabolism of xenobiotics by cytochrome P450” (**Figure 8**).

**Figure 9:**
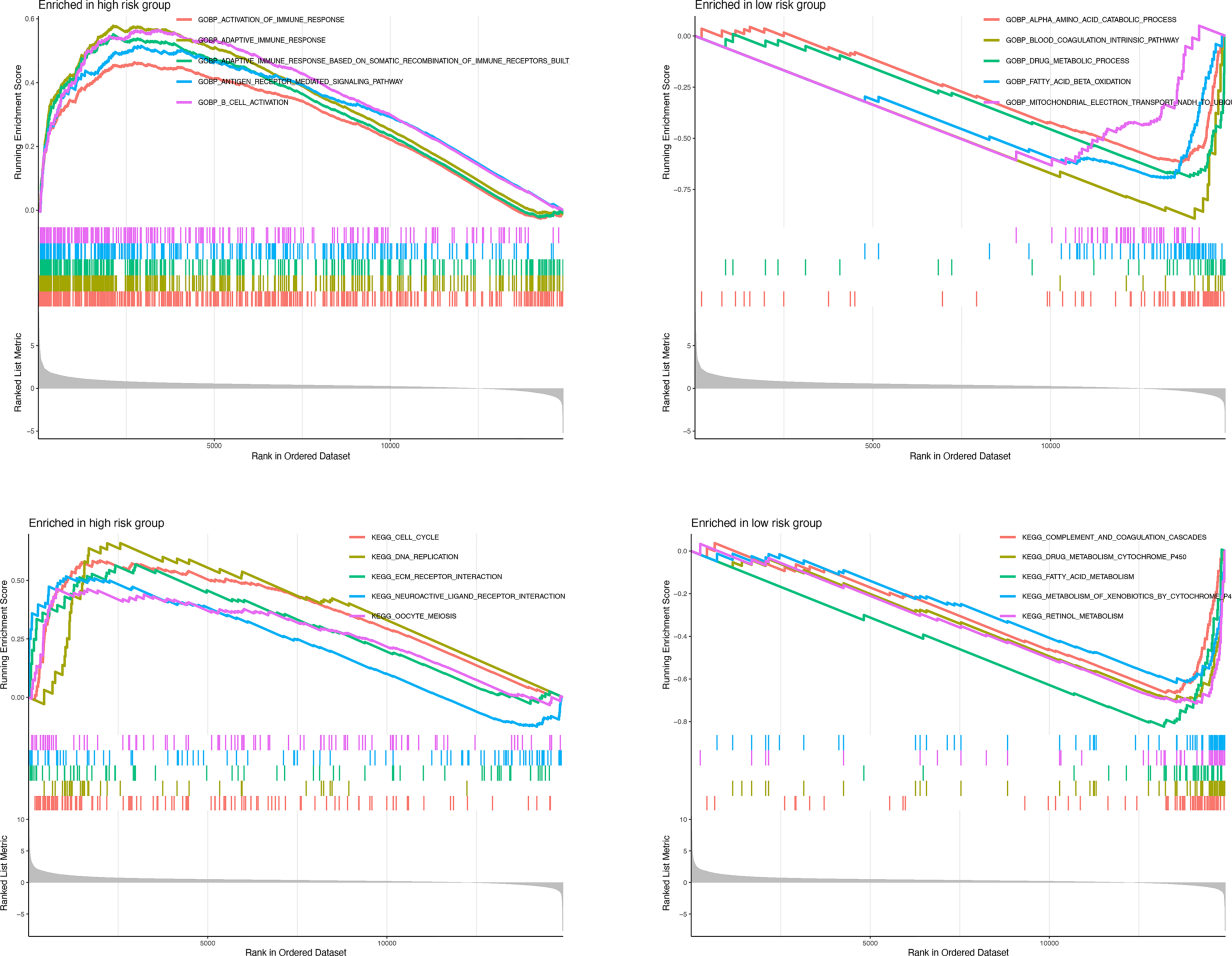
Enrichment plots from gene set enrichment analysis in the high-risk groups and low-risk groups according to risk score based on macrophage-related clusters.

GSEA was further performed to complement and validate the functional annotation of KEGG and GO. KEGG enrichment analysis indicated that the most enriched pathways in the high-risk group were “cell cycle,” “DNA replication,” “ECM receptor interaction,” and “neuroactive ligand-receptor interaction.” In contrast, “fatty acid metabolism,” “retinol metabolism,” and “drug metabolism cytochrome P450” were enriched in the low-risk groups. Additionally, GO enrichment analysis indicated that the most enriched biological processes in the high-risk group were closely linked to “humoral immune response mediated by circulating immunoglobulin,” “phagocytosis recognition,” “B-cell receptor signaling pathway,” and “positive regulation of B-cell activation” (**Figure 9**).

### Analysis of Tumor Mutation Burden in Different Risk Groups

**Figure 10:**
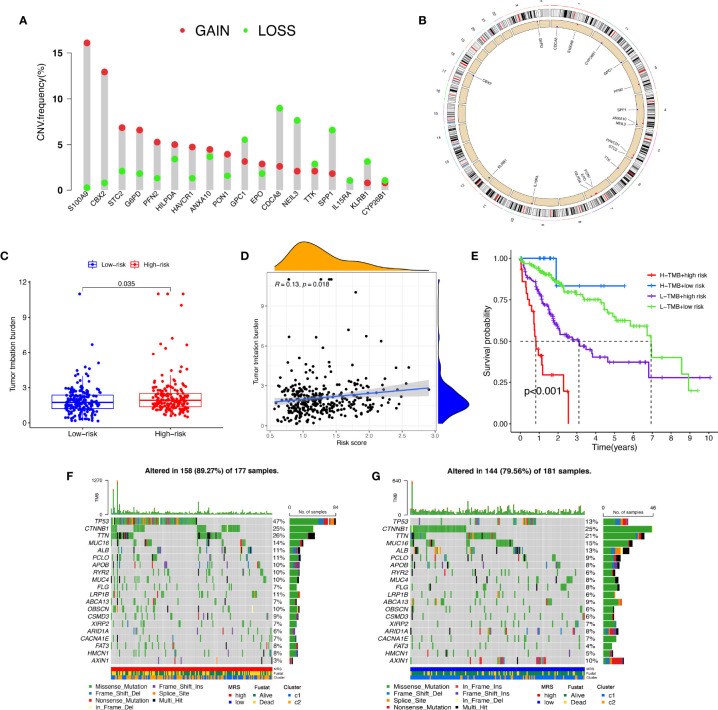
Tumor mutation analysis between different MRS signature groups. **(A)** Circus plots of chromosome distributions of selected genes from the MRS signature. **(B)** Frequencies of gain and loss for selected genes from the MRS signature. **(C)** Box plot showing tumor mutation burden in the high- and low-risk groups. **(D)** Risk scores from the MRS signature are correlated with TMB score in the TCGA dataset. **(E)** The Kaplan–Meier curve survival analysis for HCC patients stratified by both TMB groups and MRS signature. Waterfall plot displaying gene mutations in the high- **(F)** and low-risk **(G)** groups.

A CNV is a DNA fragment with copy sizes ranging from 1 kB to 1 MB in the human genome that is related to activation of oncogenes or inactivation of tumor suppressor genes and heterogeneity of the genome and molecular phenotype, further leading to the tumorigenesis and progression of tumors (28, 29). We analyzed the frequencies of genetic amplification and deletion of selected genes from the MRS. The above results revealed that *S100A9*, *CBX2*, *STC2*, *G6PD*, and *PFN2* had a higher frequency of gain-of-function mutations in HCC, while *CDCA8*, *NEIL3*, *IL15RA*, *SPP1*, and *KLRB1* had a higher frequency of loss-of-function mutations (**Figures 10A, B****)**. In view of the important role that TMB may play in clinical practice, we attempted to understand the intrinsic association between TMB and MRS. The results revealed that TMB was higher in the high-risk group and that the risk score was positively correlated with the TMB score in HCC (*r*=0.13, *P*=0.018, **Figures 10C, D****)**. Furthermore, it appeared that there was a combined influence of TMB and MRS on survival outcomes in patients with HCC (**Figure 10E**). Waterfall plots demonstrated the mutation differences of the top 20 genes between different risk groups in HCC (**Figures 10F, G****)**. Consistent with the TMB score, patients in the high-risk group had a higher mutation frequency. The mutation frequency of *TP53* in the high-risk group was significantly higher than that in the low-risk group, while the mutation frequency of *AXIN1* in the low-risk group was significantly higher, and it was mainly composed of frameshift deletion and nonsense mutation.

### The Role of MRS in Immunotherapy

**Figure 11:**
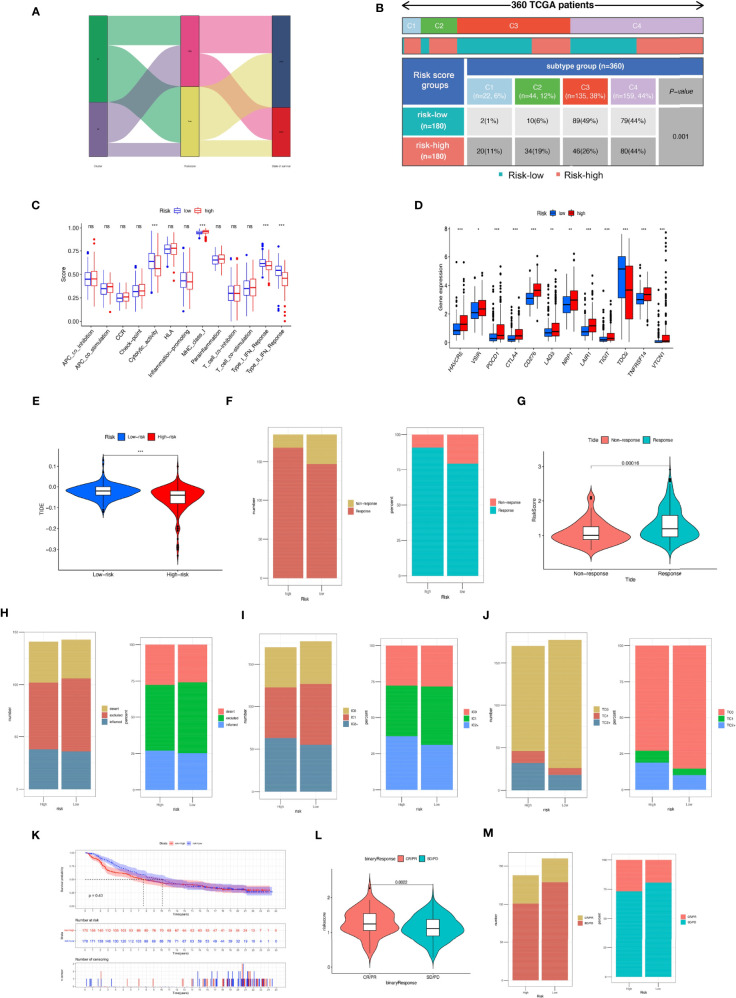
Comparison of the risk groups in our study with stage and existing immune subtype and prediction of response to immunotherapeutic agents for different risk groups. **(A)** The Sankey diagram revealed the potential connection between cluster, risk score, and survival status. **(B)** Comparison of the differences in immune subtype between different risk groups. **(C)** The boxplot illustrating the difference in immune-related functions between the high-risk and low-risk groups. **(D)** The boxplot displaying the difference in immune checkpoint genes between different cluster groups. **(E)** Comparison of the tumor immune dysfunction and exclusion (TIDE) prediction scores in the low- and high-risk groups. **(F)** Distribution and percentage of immunotherapy response among risk groups of HCC patients. **(G)** Comparison of risk scores between the response group and the non-response group. Predictive value of risk score for immunotherapy response in the IMvigor210 cohort. **(H)** Distribution and percentage of immune subtypes among risk groups of HCC patients. **(I)** Distribution and percentage of immune cell **(IC)** level type among risk groups of HCC patients. **(J)** Distribution and percentage of tumor cell (TC) level type among risk groups of HCC patients. **(K)** The Kaplan–Meier curve survival analysis between the high- and low-risk groups in the IMvigor210 cohort. **(L)** Comparison of risk scores between the CR/PR group and the SD/PD group. **(M)** Distribution and percentage of immune response type among risk groups of HCC patients in the IMvigor210 cohort. Specimens were scored as immunohistochemistry IC0, IC1, IC2, or IC3 if <1%, ≥1% but <5%, ≥5% but <10%, or ≥10% of IC were PD-L1 positive, respectively. Specimens were scored as immunohistochemistry TC0, TC1, TC2, or TC3 if <1%, ≥1% but <5%, ≥5% but <50%, or ≥50% of TC were PD-L1 positive, respectively. ns, not significant; *P < 0.05; **P < 0.01; ***P < 0.001.

We compared the proportions of immune subtypes of HCC between different risk groups. The results indicated that there were significant differences in immunophenotyping between different groups (**Figures 11A, B**). Immune-related functions, including cytolytic activity, type I IFN response, and type II IFN response, were enriched in the low-risk group, and MHC class I responses were enriched in the high-risk group. We found that the expression of ICI genes in the high-risk group was generally higher than that in the low-risk group, except for *TDO2* (**Figures 11C, D**). Additionally, the results revealed the correlation between MRS and ICI gene expression, as shown in **Supplementary Figure S6**.

We further evaluated whether the MRS could serve as an immunotherapy predictor for HCC patients. Based on the TIDE algorithm, we observed that there was a negative correlation between TIDE score and risk score, and the TIDE score in the high-risk groups was significantly lower than that in the low-risk group (**Supplementary Figure S7**). The percentage of responses in the high-risk group was higher than that in the low-risk group (**Figures 11E–G**). In the IMvigor210 cohort, 348 patients with metastatic urothelial cancer treated with anti-PD-L1 drugs were segmented into high- and low-risk groups, although no significant difference in OS was detected (*P* = 0.43), and the high-risk group showed a better survival trend (**Figure 11K**). There was no significant difference in the proportion of inflammatory immune subtypes or tumor-infiltrating immune cells expressing PD-L1 between the high- and low-risk groups (**Figures 11H, I**). However, compared with the low-risk groups, we found that the tumor tissue samples of the high-risk group had a higher proportion of tumor cells expressing PD-L1 (**Figure 11J**). In addition, we found that neoantigen count was positively correlated with risk score (**Supplementary Figure S7**), suggesting that a higher level of immune events might exist in patients with higher risk scores (*R* = 0.31, *P* = 6e−17). Compared with the low-risk group, the high-risk group had a higher percentage of complete response/partial response (**Figures 11L, M****)**. Analysis of two cell line data (BNL-MEA) from the TISMO database showed upregulated *NEIL3*, *CBX2*, and *CDCA8* related to a poor response for ICB and downregulated *NEIL3*, *CBX2*, and *CDCA8* related to a better response for ICB when compared with maintaining baseline expression level. In addition, the data revealed the downregulated *TTK*, *S100A9*, *PON1*, *PFN2*, and *GPC1* related to a poor response for ICB and upregulated *TTK*, *S100A9*, *PON1*, *PFN2*, and *GPC1* related to a better response for ICB when compared with maintaining baseline expression level (**Supplementary Figure S8**).

### Drug Sensitivity of MRS in HCC

**Figure 12:**
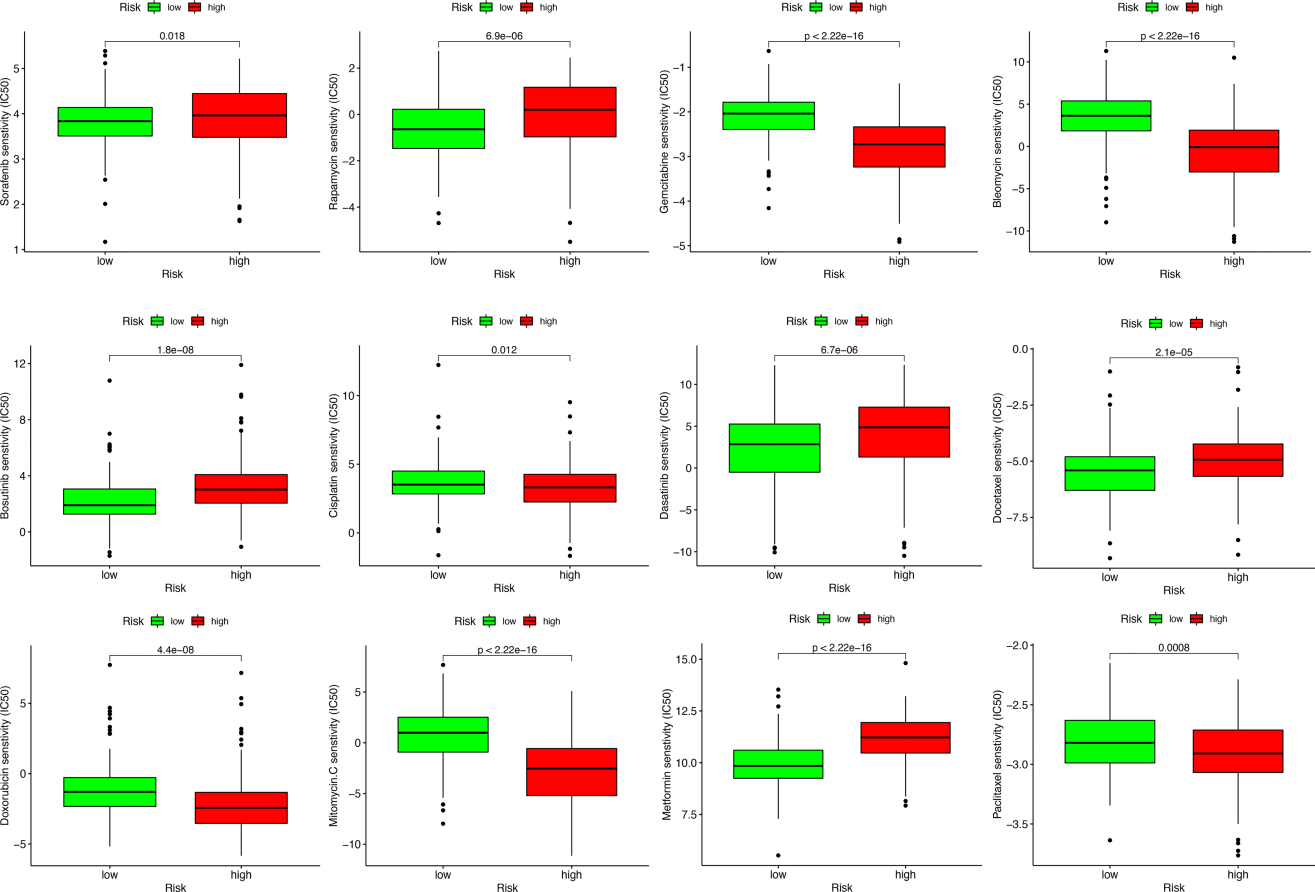
Sensitivity analysis of chemotherapeutic drugs between different risk groups.

To study the possible application of MRS in the personalized treatment of HCC, we assessed the IC_50_ values of several chemotherapy agents between the different MRS signature groups. The results of drug susceptibility indicated that the high-risk group had lower IC_50_ values of gemcitabine, bleomycin, cisplatin, doxorubicin, mitomycin C, and paclitaxel than the low-risk group, which implied that patients with high risk may benefit more from the above chemotherapies, while the IC_50_ values of sorafenib, rapamycin, bosutinib, dasatinib, docetaxel, and metformin were lower in the low-risk group, which suggested that patients with low risk may benefit more from the above chemotherapies (**Figure 12**).

## Discussion

HCC is one of the most fatal malignancies with a poor prognosis worldwide. Recently, the incidence of HCC has gradually increased due to the prevalence of viral hepatitis, alcoholism, and non-alcoholic steatohepatitis, especially in non-traditionally high-prevalence regions, such as the United States and Europe (2). Currently, HCC is treated mainly through surgical liver resection and liver transplantation, but most HCC patients are in an advanced stage when they are diagnosed, and only a minority of malignancies can be completely removed through surgery. The lack of safe and effective treatments for advanced HCC will lead to the rapid development and metastasis of the disease as well as an increase in mortality (30). HCC is a highly immunogenic malignant tumor that is characterized by a large number of immune cells around it. In recent years, as we have better understood the immunopathological mechanism and microenvironment of HCC, immunotherapy based on the regulation of the TIME has emerged as a new treatment option for HCC patients. Its clinical advantages include triggering a systemic, durable, and efficient antitumor immune response and having fewer side effects, low tumor recurrence rate, and even complete remission of some advanced tumors (31, 32). Previous studies found that macrophages, which are innate immune effector cells, play a crucial role in immunotherapy and are related to the response to immune checkpoint blockade (33, 34). It was reported that IL-6 secreted by TAMs can upregulate the expression of CD47 in HCC cells through the STAT3 signaling pathway, which in turn affects TAM-mediated phagocytosis, resulting in poor OS and recurrence-free survival (35). It was also found that M1 macrophages can promote the expression of PD-L1 on HCC cells through IL-1β, thereby promoting the development of HCC (36).

Therefore, we quantified the abundance of macrophages in the three cohorts, then systematically explored the expression of macrophage-related genes that influenced the prognosis of HCC patients and identified a hub module of genes related to macrophage infiltration level in HCC. We used the unsupervised clustering to divide HCC into two molecular subtypes based on these genes: cluster 1 and cluster 2. We observed that the prognosis of macrophage-related cluster 2 was significantly worse than that of cluster 1. Cluster 2 had higher stromal and immune scores and its ICI gene expression was also higher, but tumor purity was lower. The high expression of the ICI gene is more likely to foster an immunosuppressive microenvironment and facilitate tumor immune escape. We observed that cluster 1 was mainly enriched in metabolism-related pathways, while cluster 2 was mainly related to immune pathways. In addition, cluster 2 is more responsive to anti-PD-1 and anti-CTLA4 treatments, which indicates that cluster 2 is an immune-favorable tumor.

In order to better apply these subtypes of macrophage clusters to the clinical treatment of HCC and calculate specific macrophage-related risk scores for each HCC patient, we explored the DEGs between the two clusters and used LASSO Cox regression analysis to establish an MRS model to quantify the prognostic risk based on the two clusters and to provide promising prognostic biomarkers of HCC that could predict the response to various chemotherapies and immunotherapies. Among the genes identified by LASSO analysis, *IL15RA* usually exists in heterotrimeric receptors together with *IL2RB* and *IL2RG* and binds to IL15 with high affinity to activate signal transduction, which is associated with the regulation of the body’s adaptive immune response and the activation and maintenance of different lymphocyte populations (37–39). Studies have reported that the coexpression of *IL15RA* and IL15 in breast cancer cell lines can promote tumor cancer proliferation, prevent tumor cell apoptosis, and enhance cell migration. Studies have reported that the coexpression of *IL15RA* and IL15 in breast cancer cell lines can promote cancer proliferation, prevent cell apoptosis, and enhance cell migration (37). A recent study demonstrated that NEIL3 could repair oxidative DNA damage at telomeres in mitosis, thereby preventing the senescence of HCC cells (40). Similarly, the high expression of HILPDA in tumor cells may contribute to TAM infiltration and is related to tumor immunosuppressive status (41). EPO acts on the EPO receptors on the surface of tumor cells to increase the suppression of T cells in the immune microenvironment mediated by macrophages (42). *S100A9*, which is an immunosuppressive TAM marker, is related to the shorter survival period of cancer patients and adverse reactions to immunotherapy (43). *S100A9* can increase TAM infiltration by promoting CCL2 secretion and enhance the stem cell-like characteristics of HCC cells through Ca^2+^-dependent signal transduction of the AGER/NF-κB axis (44). Many other genes in the MRS model, such as *PFN2*, *HAVCR1*, *CDCA8*, *CYP26B1*, *TTK*, *SPP1*, *STC2*, and *CBX2* play vital roles in regulating and participating in the progression of different cancers, thereby affecting tumor cell proliferation, migration, and invasion and epithelial–mesenchymal transition (45–52). *ANXA10* is a calcium-/phospholipid-binding protein. Previous studies found inconsistent effects of *ANXA10* on tumors, and there is controversy. Studies have reported that the expression of *ANXA10* is significantly increased in melanoma and can promote melanoma metastasis by suppressing E3 ligase TRIM41-directed PKD1 degradation (53). However, in gastric cancer, *ANXA10* plays a role in suppressing cancer (54). The *KLRB1* gene encodes the CD161 receptor of natural killer cells, thereby regulating the cytotoxic function of cells and regulating the production of cytokines (55). However, in a recent study, it was found that CD8^+^ T cells overexpressed *KLRB1* in HCC with early recurrence and showed a congenital state of low cytotoxicity, which affected the prognosis of HCC patients (56). More in-depth research is needed to explore the mechanism of *ANXA10* and *KLRB1* in HCC.

CNVs can facilitate cancer progression by activating or inactivating oncogenes. It has been reported that frequent CNVs in cancer cells increase tumor heterogeneity (57). We found that the oncogenic driver genes *S100A9* and *CBK2* had copy number amplification, and the change in copy number might change the immune infiltration state of the body to a certain extent. TMB is thought to be a predictive biomarker of tumor biological behavior and immune response, and the accumulation of somatic mutations is one of the main causes of tumorigenesis and facilitates the expression of neoantigens (58). Our analysis indicated that there was a significant difference in the survival time of patients in the high- and low-TMB groups. We found that the prognosis of HCC patients can be effectively distinguished based on TMB and MRS. Additionally, we found that the frequency of *TP53* gene mutations was higher in the high-risk group, while the *AXIN1* mutation rate was higher in the low-risk group. *TP53* is a commonly mutated gene in tumors, playing a vital role in regulating cell stress, DNA damage repair, and cell apoptosis and inhibiting the body’s immune response (59). *TP53* could act as a physiological brake on the M2 macrophage polarization process through the *TP53*/MDM2/c-MYC axis, and its mutation has been confirmed to be significantly related to the poor prognosis of many tumors (60). There was no significant difference in the somatic activating mutation frequency of the *CTNNB1* gene coding for β-catenin between the high- and low- risk groups. *AXIN1* is a negative regulator of the Wnt/β-catenin signaling pathway (61), but whether the *AXIN1* mutation causes Wnt/β-catenin activation is still controversial. Studies have shown that *CTNNB* mutation status was related to the upregulation of Wnt/β-catenin pathway genes instead of *AXIN1* mutation and *AXIN1* mutation was unlikely to be a strong driving factor in the development of HCC in humans (62), which may explain why the low-risk group had a better prognosis despite the higher frequency of *AXIN1* mutations.

Immunotherapy related to ICIs is a promising method to treat a variety of malignant tumors, and we found that there was a positive correlation between the risk score of the MRS model and almost all immune checkpoint gene expression levels. Similarly, it is worth noting that the proportions of the C1 subtype (wound healing) and C2 subtype (IFN-γ dominant) in the high-risk group were significantly higher, and the proportion of the C3 subtype (inflammatory) was significantly lower. It was reported that the C2 subtype (IFN-γ dominant) was related to the polarization of M1/M2 macrophages and can lead to increased tumor cell proliferation, which may override an evolving type I immune response; however, the C3 subtype, which has obvious Th17 characteristics, might represent immunologic control of the disease. The scatter plot revealed a negative correlation between the MRS score and TIDE score in the TCGA cohort, and a higher MRS model score might be more likely to benefit from immunotherapy. Previous studies have reported that cross-tumor information could be used to predict the effect of immunotherapy (63, 64). Herein, to test the effectiveness of the MRS model in distinguishing immunotherapy outcomes, we used the IMvigor210 cohort of 348 patients with urothelial cancer to test the predictive value of immunotherapy of the MRS models for immunotherapeutic effect. We observed that the high-risk group had a higher proportion of complete remission and partial remission after immunotherapy. There was no significant difference in survival between the two groups, which might be related to the survival benefits of immunotherapy for high-risk patients. The above results verify the conclusion that the MRS model might be used as an immunotherapy indicator. Notably, the sensitivity of HCC to various chemotherapeutic drugs is relatively poor due to the existence of drug resistance mechanisms and heterogeneity, resulting in limited benefit from chemotherapy. In our study, different risk groups had different responses to traditional chemotherapy drugs, which indicated that the MRS model we established could also assist in the choice of chemotherapy drugs for HCC patients.

This study aimed to divide HCC patients into different macrophage cluster subtypes, identify DEGs between different clusters, and establish an MRS model and link macrophage-related genes with the prognosis of HCC patients. We have performed multiangle and multidatabase validation, and the MRS signature model shows good prospects in predicting the prognosis of HCC patients. Our study still has some limitations. First, the clinical data we downloaded from public databases are incomplete and lack some important clinical details, such as AFP, range of liver resection scope, and microvascular infiltration, so we could not explore the impact of the above factors on the prognosis of HCC patients. We need to conduct a prospective, multicenter study with a larger sample to verify the accuracy of the MRS model we established. In addition, the results of single-cell sequencing can help us better understand the changes in macrophage-related genes in the HCC tumor microenvironment. Second, functional experiments (both *in vitro* and *in vivo*) should be performed to further clarify the molecular mechanism through which macrophage-related genes affect HCC.

In conclusion, this study identified macrophage-related genes in HCC patients; furthermore, we established and validated the MRS model to predict the OS of HCC patients, and it showed good predictive ability. We also assessed the differences in immunotherapy response and chemotherapeutic drug sensitivity between MRS risk groups. The above results may help to advance our understanding of the features of macrophage infiltration and provide new strategies for personalized therapy.

## Data Availability Statement

The datasets presented in this study can be found in online repositories. The names of the repository/repositories and accession number(s) can be found in the article/**Supplementary Material**.

## Ethics Statement

Written informed consent was obtained from the individual(s) for the publication of any potentially identifiable images or data included in this article.

## Author Contributions

Study concept and design: TW, LD, SS, and WW. Acquisition of data and statistical analysis of data: TW, YY, and MY. Drafting of the manuscript: TW and LD. Critical revision of the manuscript: YQ and WW. All authors contributed to the article and have approved the submitted manuscript.

## Funding

This research was supported by the Science and Technology Program of Sichuan Science and Technology Department (Nos. 2019YFS0029, 2019YFS0529), the National Natural Science Foundation of China (Nos. 81770566, 82170543, 82000599) and the New Medical Technology Foundation of West China Hospital of Sichuan University (No. XJS2016004).

## Conflict of Interest

The authors declare that the research was conducted in the absence of any commercial or financial relationships that could be construed as a potential conflict of interest.

## Publisher’s Note

All claims expressed in this article are solely those of the authors and do not necessarily represent those of their affiliated organizations, or those of the publisher, the editors and the reviewers. Any product that may be evaluated in this article, or claim that may be made by its manufacturer, is not guaranteed or endorsed by the publisher.

## Supplementary Material

Supplementary Figure 1 Expressions of the 69 differentially expressed macrophage co-expression genes between tumor tissues and normal samples and their interactions. **(A)** The heatmap showing 69 differentially expressed macrophage co-expression gene between tumor tissues and normal samples (blue: low expression level; red: high expression level). **(B)** PPI network showing the interactions of the macrophage-related differentially expressed genes (interaction score = 0.9). **(C)** The correlation network analysis of the macrophage-related candidate genes (red line: positive correlation; blue line: negative correlation. The intensity of the color reflects the degree of its relevance).

Supplementary Figure 2 Coefficients for each gene in MRS signature based on the macrophage-related clusters by the LASSO regression algorithm.

Supplementary Figure 3 Kaplan–Meier survival curves for selected genes which obtained by LASSO Cox regression analysis.

Supplementary Figure 4 Verifying protein expression levels of genes from MRS model in normal liver tissue and HCC using the HPA database.

Supplementary Figure 5 MRS signature based on macrophages related clusters was associated with the clinicopathological characters of patients with HCC in the GSE14520 dataset and ICGC dataset. **(A)** Heatmap for MRS signature based on macrophages related clusters and clinicopathological manifestation in the GSE14520 dataset. **(B)** Boxplot of risk score based on macrophages related clusters in HCC patients with different size. **(C)** Boxplot of risk score based on macrophages related clusters in HCC patients with different AFP. **(D)** Boxplot of risk score based on macrophages related clusters in HCC patients with different multinodular status. **(E)** Boxplot of risk score based on macrophages related clusters in HCC patients with different stage. **(F)** Boxplot of risk score based on macrophages related clusters in HCC patients with different cirrhosis status. **(G)** Boxplot of risk score based on macrophages related clusters in HCC patients with different status of survival. **(H)** Heatmap for MRS signature based on macrophages related clusters and clinicopathological manifestation in the ICGC dataset. **(I)** Boxplot of risk score based on macrophages related clusters in HCC patients with different stage. **(J)** Boxplot of risk score based on macrophages related clusters in HCC patients with different status of survival.

Supplementary Figure 6 **(A)** Chord diagram illustrating the correlations between the expression of 15 key immune checkpoint genes, as well as between the mRNA expression of 15 key immune checkpoint and the risk score from the MRS signature in TCGA-LIHC dataset. Association between risk score from the MRS signature and **(B)** BTNL2, **(C)** CD274, **(D)** CD276, **(E)** CTLA4, **(F)** HAVCR2, **(G)** IDO1, **(H)** LAG3, **(I)** LAIR1, **(J)** NP1, **(K)** PDCD1, **(L)** TDO2, **(M)** TIGHT, **(N)** TNFRSF14-AS1, **(O)** VSIR, **(P)** VTCN1.

Supplementary Figure 7 Association between risk score from the MRS signature and **(A)** TIDE score and **(B)0** neoantigen burden per MB.

Supplementary Figure 8 Gene expression levels across groups of different responses to ICBs in syngeneic mouse models.
