# Supplementary figures and images for: Comprehensive Molecular Analyses of a Macrophage-Related Gene Signature With Regard to Prognosis, Immune Features, and Biomarkers for Immunotherapy in Hepatocellular Carcinoma Based on WGCNA and the LASSO Algorithm
Source: Front Immunol. 2022 May 27;13:843408. doi: 10.3389/fimmu.2022.843408 (PMC9186446; doi:10.3389/fimmu.2022.843408)

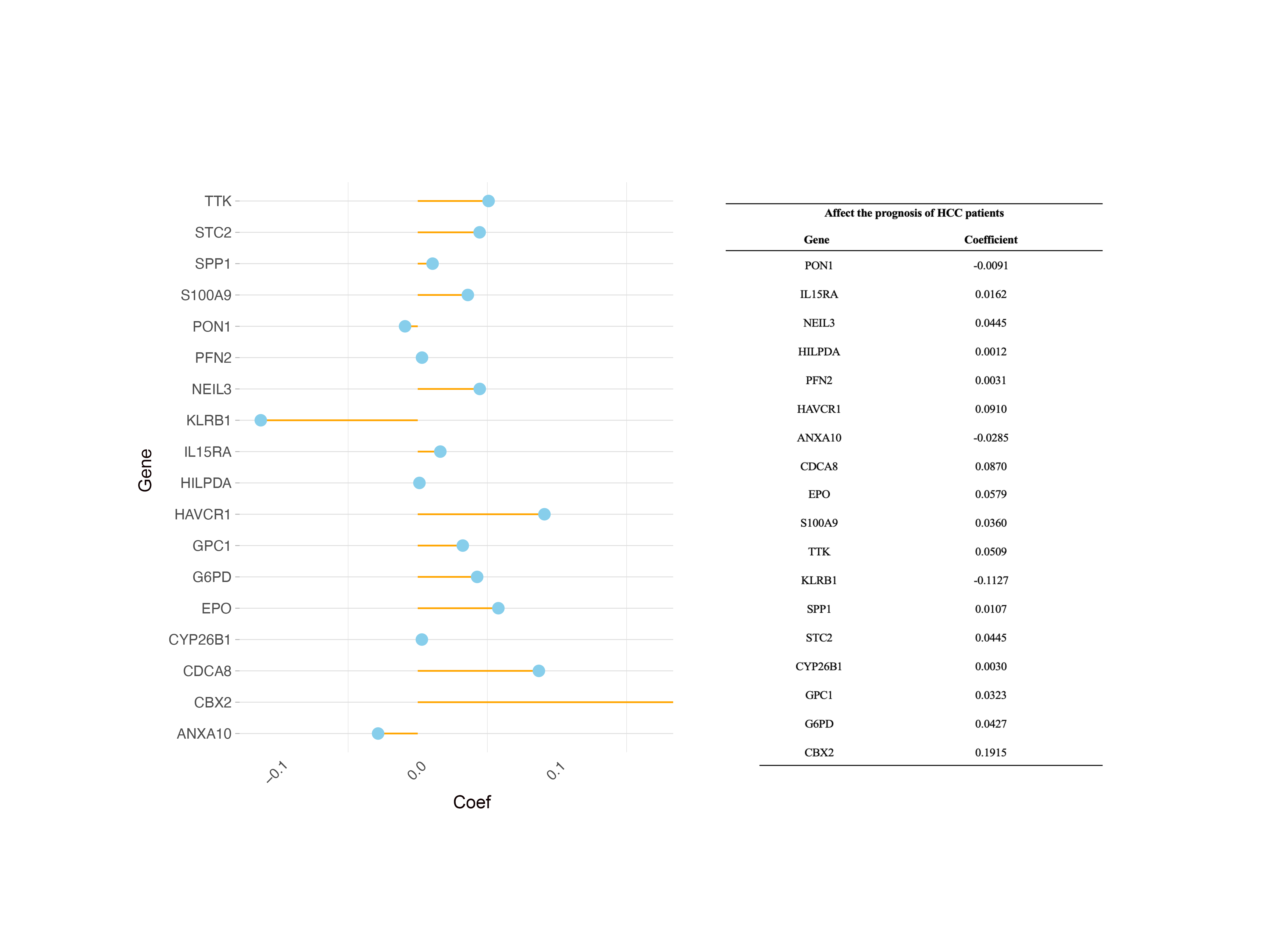

Supplement: Supplementary Figure 2 — Coefficients for each gene in MRS signature based on the macrophage-related clusters by the LASSO regression algorithm. [file Image_2.tif]

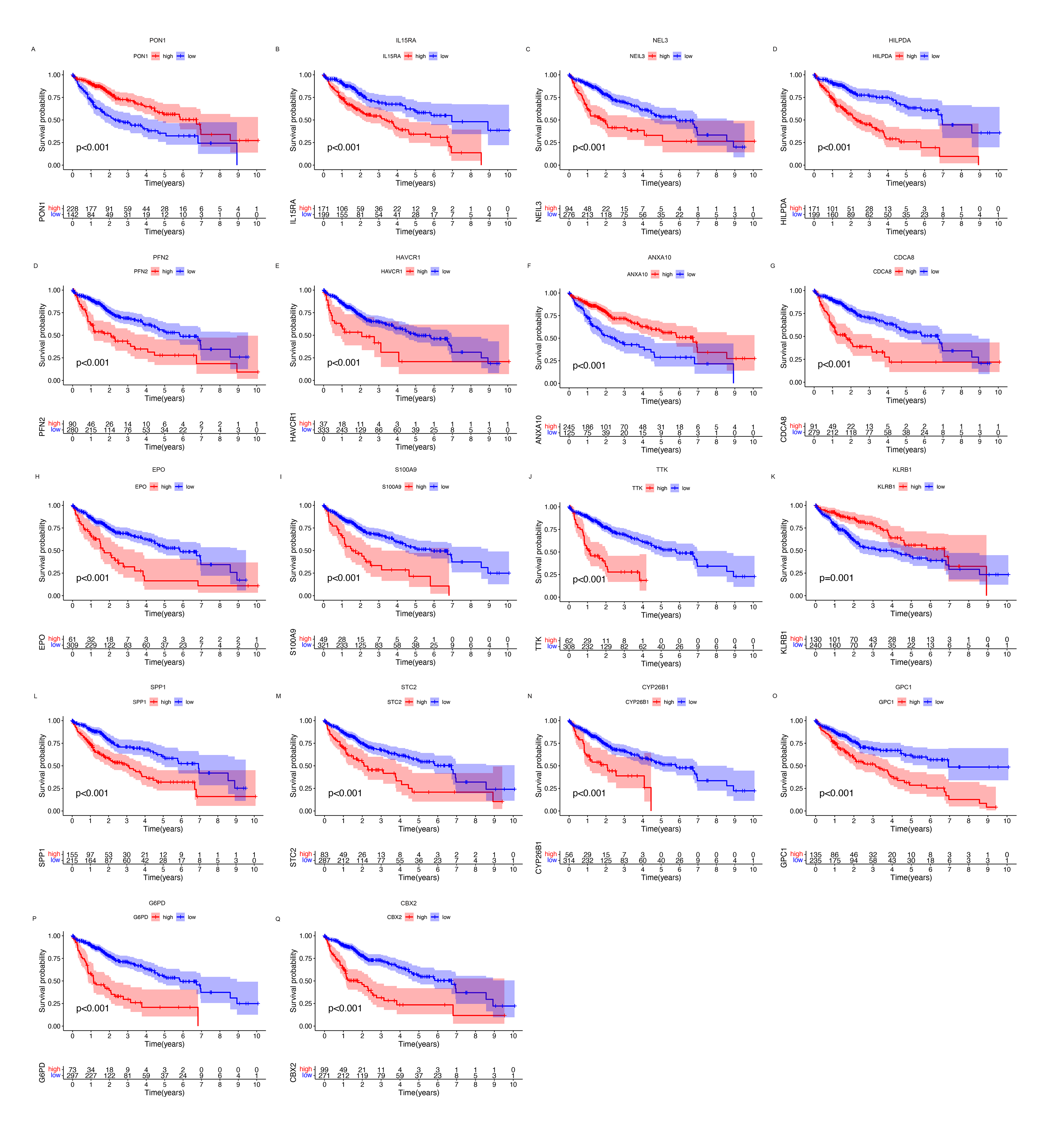

Supplement: Supplementary Figure 3 — Kaplan–Meier survival curves for selected genes which obtained by LASSO Cox regression analysis. [file Image_3.tif]

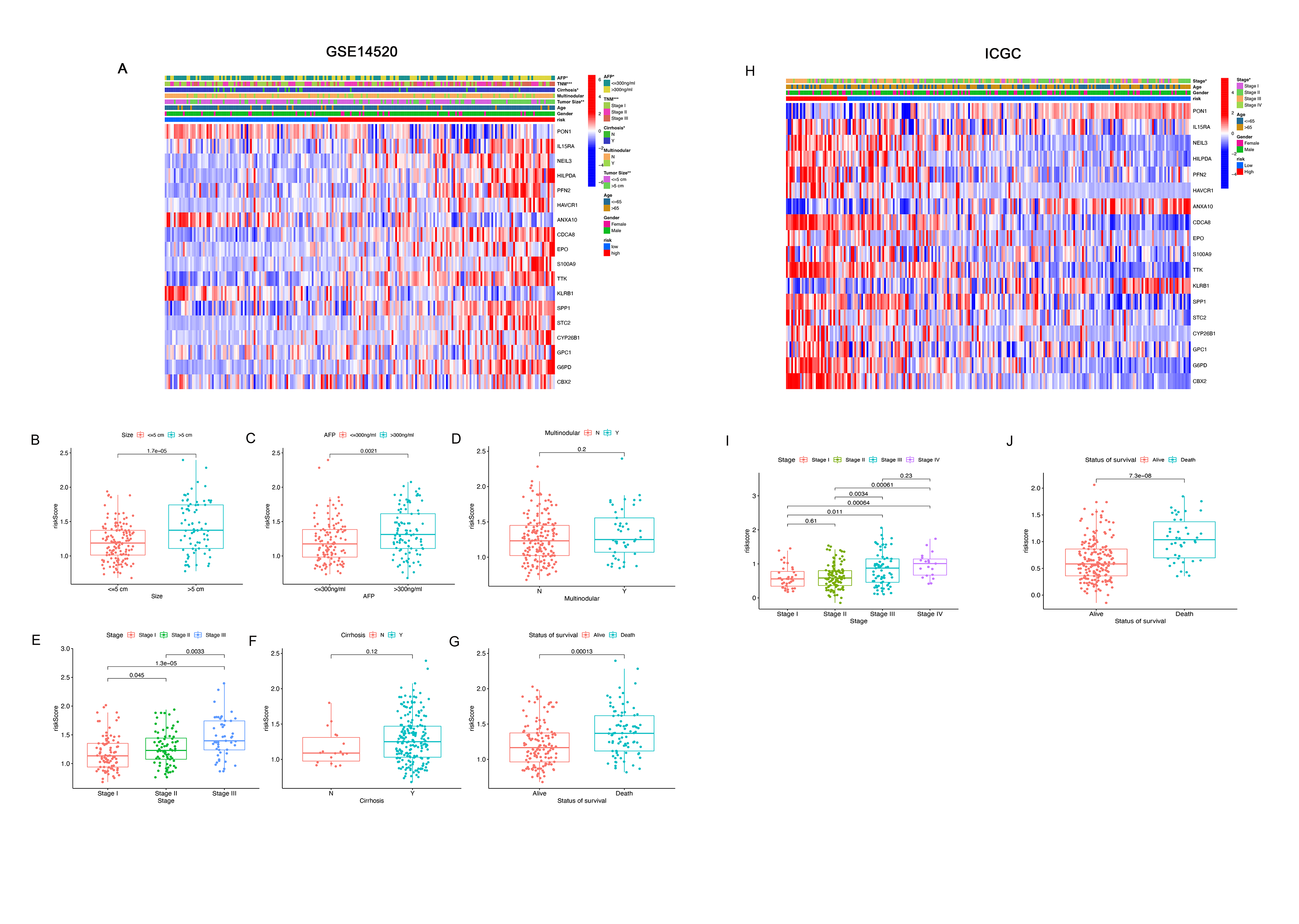

Supplement: Supplementary Figure 5 — MRS signature based on macrophages related clusters was associated with the clinicopathological characters of patients with HCC in the GSE14520 dataset and ICGC dataset. (A) Heatmap for MRS signature based on macrophages related clusters and clinicopathological manifestation in the GSE14520 dataset. (B) Boxplot of risk score based on macrophages related clusters in HCC patients with different size. (C) Boxplot of risk score based on macrophages related clusters in HCC patients with different AFP. (D) Boxplot of risk score based on macrophages related clusters in HCC patients with different multinodular status. (E) Boxplot of risk score based on macrophages related clusters in HCC patients with different stage. (F) Boxplot of risk score based on macrophages related clusters in HCC patients with different cirrhosis status. (G) Boxplot of risk score based on macrophages related clusters in HCC patients with different status of survival. (H) Heatmap for MRS signature based on macrophages related clusters and clinicopathological manifestation in the ICGC dataset. (I) Boxplot of risk score based on macrophages related clusters in HCC patients with different stage. (J) Boxplot of risk score based on macrophages related clusters in HCC patients with different status of survival. [file Image_5.tif]

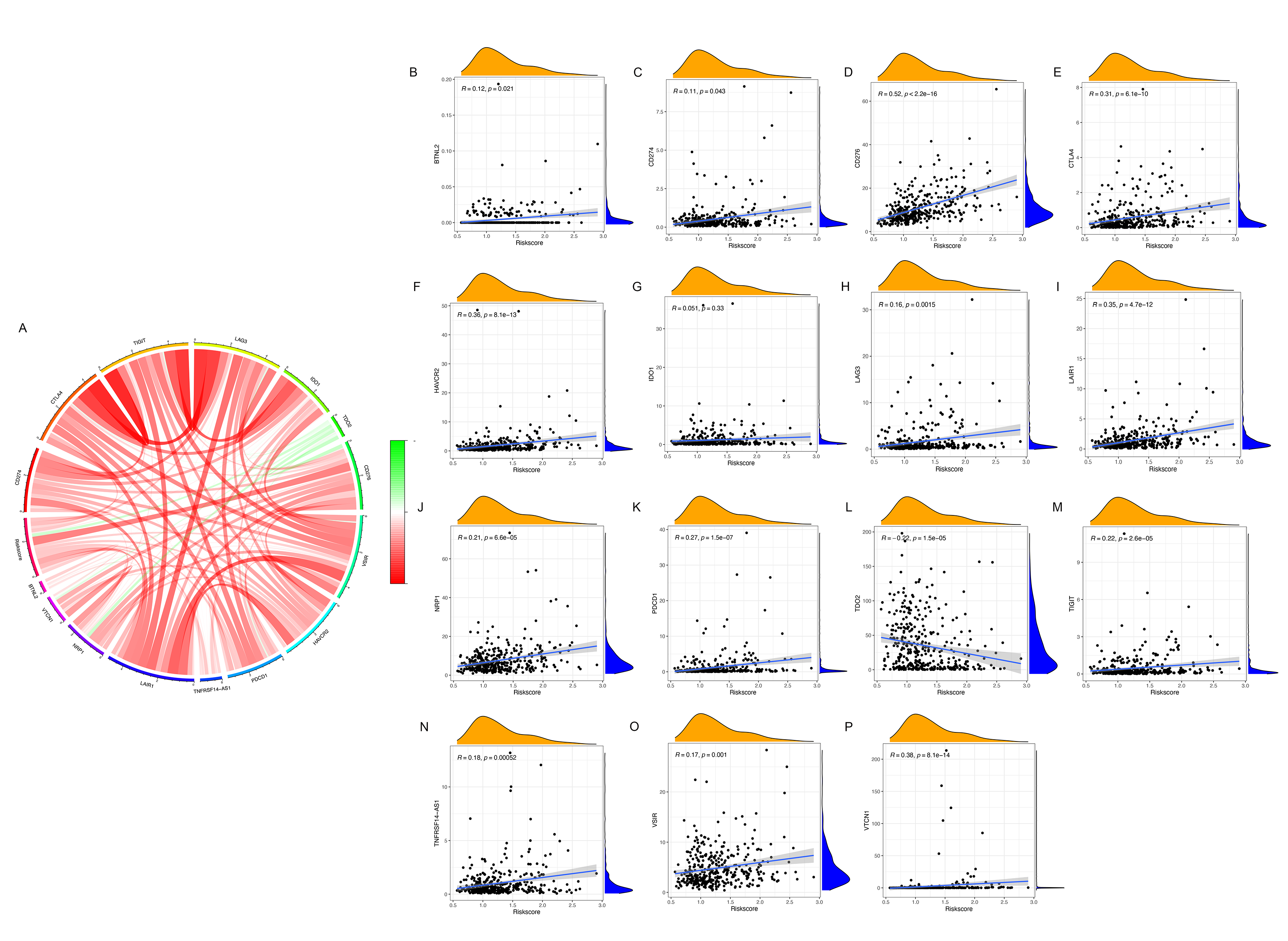

Supplement: Supplementary Figure 6 — (A) Chord diagram illustrating the correlations between the expression of 15 key immune checkpoint genes, as well as between the mRNA expression of 15 key immune checkpoint and the risk score from the MRS signature in TCGA-LIHC dataset. Association between risk score from the MRS signature and (B) BTNL2, (C) CD274, (D) CD276, (E) CTLA4, (F) HAVCR2, (G) IDO1, (H) LAG3, (I) LAIR1, (J) NP1, (K) PDCD1, (L) TDO2, (M) TIGHT, (N) TNFRSF14-AS1, (O) VSIR, (P) VTCN1. [file Image_6.tif]

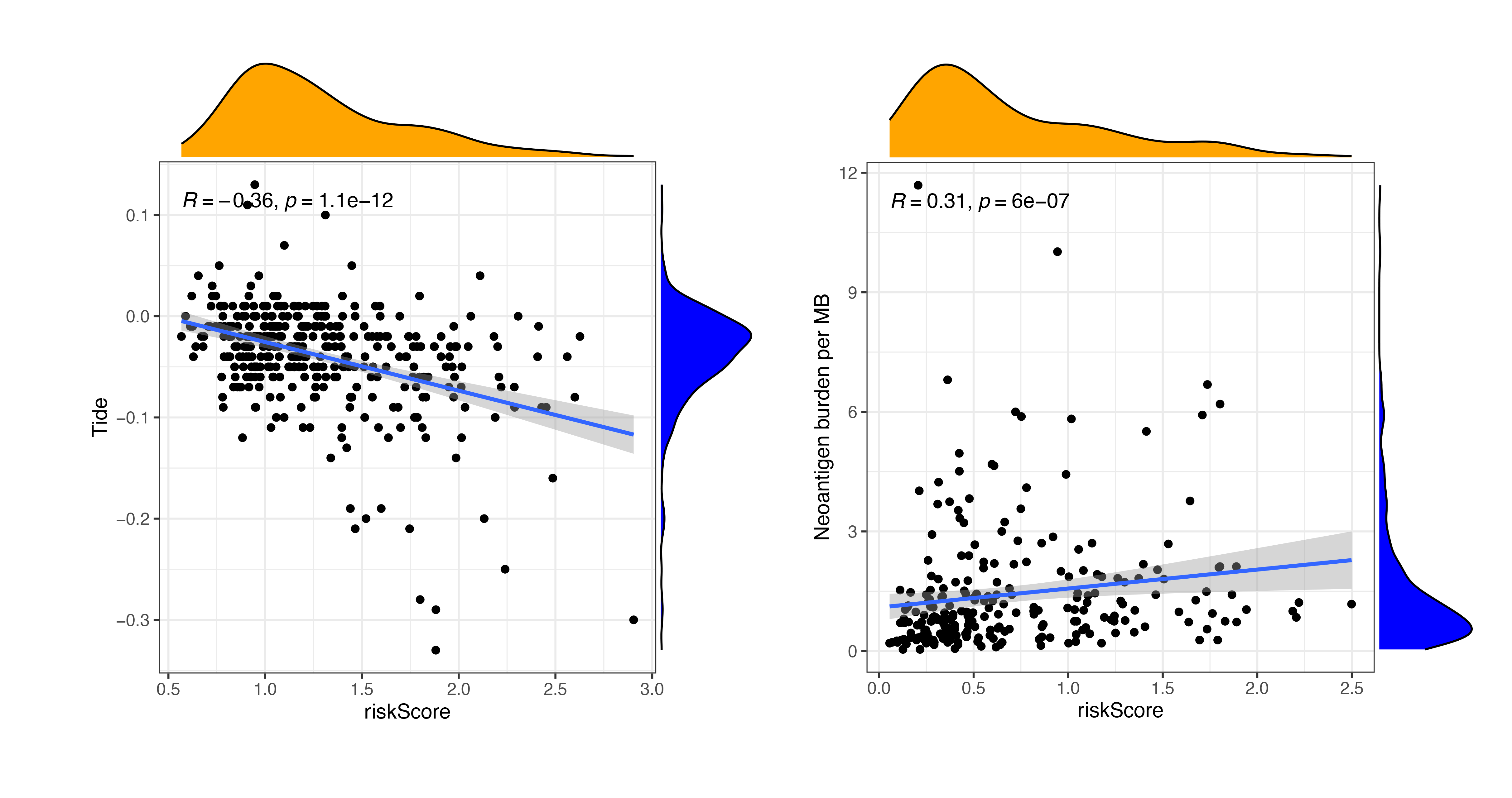

Supplement: Supplementary Figure 7 — Association between risk score from the MRS signature and (A) TIDE score and (B)0 neoantigen burden per MB. [file Image_7.tif]

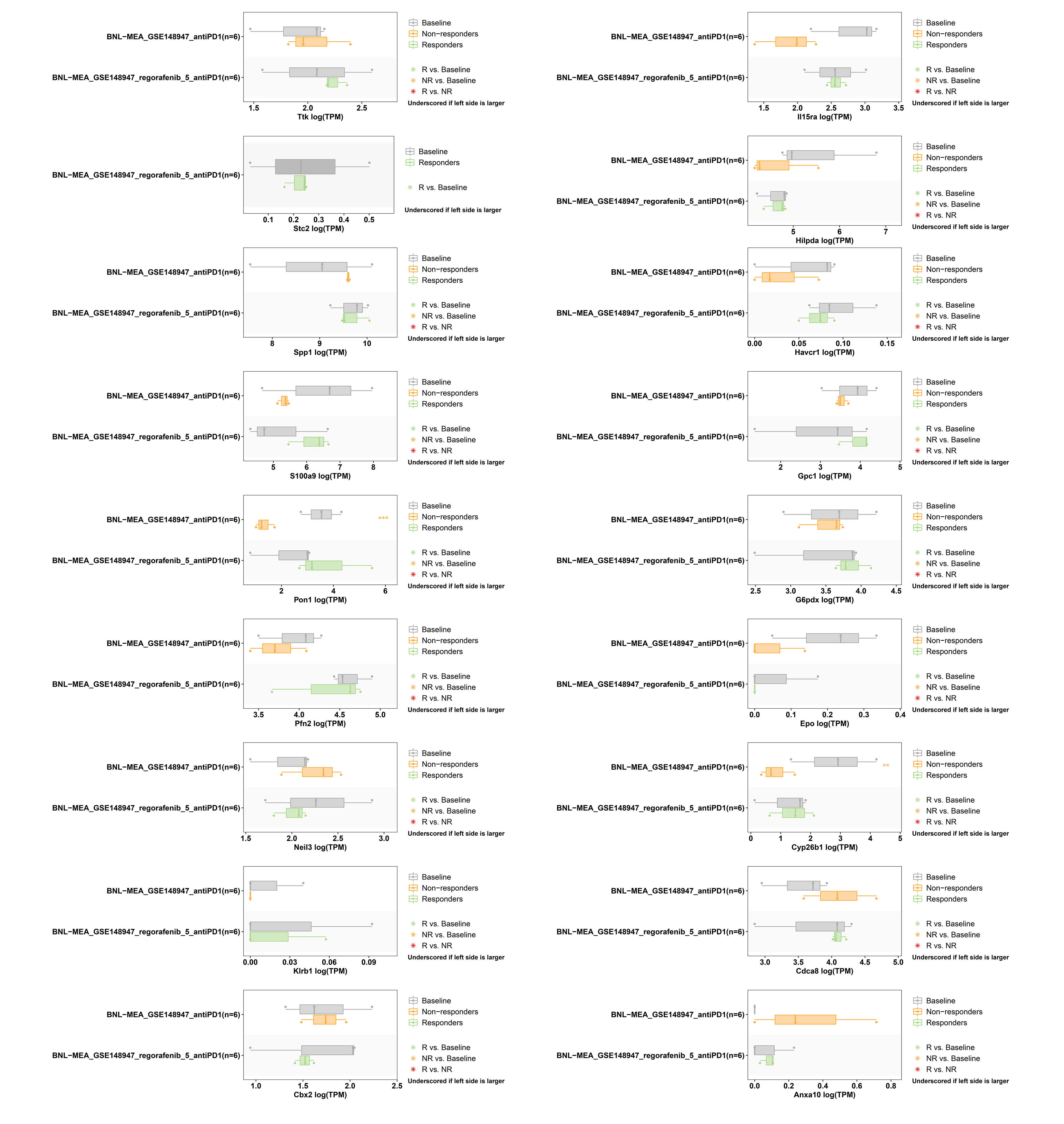

Supplement: Supplementary Figure 8 — Gene expression levels across groups of different responses to ICBs in syngeneic mouse models. [file Image_8.tif]
